# Prediction of long-term mortality by using machine learning models in Chinese patients with connective tissue disease-associated interstitial lung disease

**DOI:** 10.1186/s12931-022-01925-x

**Published:** 2022-01-07

**Authors:** Di Sun, Yu Wang, Qing Liu, Tingting Wang, Pengfei Li, Tianci Jiang, Lingling Dai, Liuqun Jia, Wenjing Zhao, Zhe Cheng

**Affiliations:** Department of Respiratory and Critical Care Medicine, The First Affiliated Hospital of Zhengzhou University, Zhengzhou, The People’s Republic of China

**Keywords:** Interstitial lung disease, Connective tissue disease, Nomogram, ILD-GAP, Machine learning, LASSO

## Abstract

**Background:**

The exact risk assessment is crucial for the management of connective tissue disease-associated interstitial lung disease (CTD-ILD) patients. In the present study, we develop a nomogram to predict 3‑ and 5-year mortality by using machine learning approach and test the ILD-GAP model in Chinese CTD-ILD patients.

**Methods:**

CTD-ILD patients who were diagnosed and treated at the First Affiliated Hospital of Zhengzhou University were enrolled based on a prior well-designed criterion between February 2011 and July 2018. Cox regression with the least absolute shrinkage and selection operator (LASSO) was used to screen out the predictors and generate a nomogram. Internal validation was performed using bootstrap resampling. Then, the nomogram and ILD-GAP model were assessed via likelihood ratio testing, Harrell’s C index, integrated discrimination improvement (IDI), the net reclassification improvement (NRI) and decision curve analysis.

**Results:**

A total of 675 consecutive CTD-ILD patients were enrolled in this study, during the median follow-up period of 50 (interquartile range, 38–65) months, 158 patients died (mortality rate 23.4%). After feature selection, 9 variables were identified: age, rheumatoid arthritis, lung diffusing capacity for carbon monoxide, right ventricular diameter, right atrial area, honeycombing, immunosuppressive agents, aspartate transaminase and albumin. A predictive nomogram was generated by integrating these variables, which provided better mortality estimates than ILD-GAP model based on the likelihood ratio testing, Harrell’s C index (0.767 and 0.652 respectively) and calibration plots. Application of the nomogram resulted in an improved IDI (3- and 5-year, 0.137 and 0.136 respectively) and NRI (3- and 5-year, 0.294 and 0.325 respectively) compared with ILD-GAP model. In addition, the nomogram was more clinically useful revealed by decision curve analysis.

**Conclusions:**

The results from our study prove that the ILD-GAP model may exhibit an inapplicable role in predicting mortality risk in Chinese CTD-ILD patients. The nomogram we developed performed well in predicting 3‑ and 5-year mortality risk of Chinese CTD-ILD patients, but further studies and external validation will be required to determine the clinical usefulness of the nomogram.

## Background

Connective tissue disease (CTD) which consists of many autoimmune mechanisms is characterized by self-directed inflammation often leading to collagen deposition, tissue damage and ultimately target organs failure [1]. CTD could involve multiple organs and systems, among which interstitial lung disease (ILD) remains a main cause of morbidity and mortality [2]. The median survival time for patients with CTD-associated ILD (CTD-ILD) was reported to be around 6.5 years, and up to 12.4% of patients with CTD-ILD die of ILD [3, 4]. Thus, the exact risk assessment is crucial for the management of CTD-ILD patients.

The risk prediction of CTD-ILD remains challenging, due to the heterogeneity in patient-specific and disease-specific variables. The ILD-gender-age-physiology (ILD-GAP) model is a multidimensional mortality risk prediction model composed by the ILD diagnosis, sex, age, the percent predicted values of forced vital capacity (FVC %Predicted) and the percent predicted values of diffusion capacity of lung for carbon monoxide (DLco %Predicted). Since the ILD-GAP model was firstly established by Christopher J. Ryerson et al. based on North America population, it was wildly used to predict mortality across all chronic ILD subtypes, including CTD-ILD [5]. However, the ILD-GAP model has not been validated in Chinese CTD-ILD patients. Therefore, more inclusive studies are needed to validate and improve the prediction accuracy of the existing assessment model.

We performed this study to establish a comprehensive predictive nomogram by using machine learning algorithms, involving demographic characteristics, clincal features, echocardiography, laboratory testing as well as imageological examination. Furthermore, we also validated whether the combination of the nomogram and ILD-GAP model could generate a superior prognostic performance.

## Methods

### Patients

CTD-ILD patients who were diagnosed and treated at the First Affiliated Hospital of Zhengzhou University were enrolled based on a prior well-designed criterion between February 2011 and July 2018. The patients would be included if they met four of the following inclusion criteria: (1) Patients were diagnosed CTD-ILD recommendated by the American Rheumatism Association and the American College of Rheumatology [6–12], including polymyositis/dermatomyositis (PM/DM), systemic lupus erythematosus (SLE), systemic sclerosis (SSc), ankylosing spondylitis (AS), sjogren syndrome (SS), mixed connective tissue disease (MCTD), rheumatoid arthritis (RA), undifferentiated connective tissue disease (UCTD) and overlap syndromes (OCTD). UCTD patients should also followed the diagnostic criteria for UCTD-ILD established by the previous research [13]; (2) having clinical symptoms (dyspnea or cough); (3) having signs suggestive of ILD (endinspiratory bibasilar crepitations); (4) having radiographic signs (honeycombing, ground-glass opacities, nodular or reticulonodular) of ILD confirmed by high-resolution computed tomography (HRCT). The patients would be excluded if they met one of the following exclusion criteria: (1) Age younger than 18 years; (2) pregnancy; (3) lossing to follow-up; (4) incomplete clinical records. This study received the Institutional Review Board approval by the First Affiliated Hospital of Zhengzhou University (2019-KY-116).

### Data collection

Demographic variables were extraction from medical chart review, including age, sex, occupation, smoking history, days of symptoms, medication treatment history, chronic disease history (diabetes and hypertension), CTD types, PFTs, echocardiography, laboratory data (routine inflammatory, hematological and biochemical parameters) and chest HRCT.

The collected PTFs data included FVC %Predicted, the percent predicted values of forced expiratory volume in one second (FEV_1_%Predicted), FEV_1_/FVC and DLco %Predicted.

The collected echocardiography data included right ventricular diameter (RVD), right atrial area (RAA), left ventricular diameter, aortic annulus diameter, left atrial diameter, ascending aortic diameter, pulmonary artery diameter, pulmonary artery systolic pressure (PASP), aortic valve regurgitation peak velocity, tricuspid regurgitant peak velocity and left ventricular ejection fraction (LVEF).

The collected laboratory data included klebs von den Lungen-6, procalcitonin, complement component C4, complement component C3, C-reactive protein (CRP), erythrocyte sedimentation rate, leukocyte count, platelet count, hemoglobin count, erythrocyte count, hematocrit, blood urea nitrogen, B-type natriuretic peptide (BNP), uric acid, creatinine, fasting blood glucose, aspartate transaminase (AST), alanine aminotransferase, γ-Glutamyltranspeptidase (GGT), alkaline phospatase, total protein, albumin (ALB), globulin, triglyceride, prothrombin time, cholesterol, activated partial thromboplastin time, prothrombin time activity, thrombin time, international normalized ratio, fibrinogen and D‐dimer.

HRCT images were reviewed independently by 2 expert thoracic radiologists, who were kept blinded for patients’ diagnosis. Images were re-evaluated till reaching a consensus when divergence occurred. The collected HRCT characteristics included honeycombing, ground-glass opacities, nodular, fine reticular opacities, local pleural thickening, pulmonary bullous, hydrothorax and hydropericardium.

### Follow‑up and study outcome

All-cause mortality was the endpoint during follow-up until July 2021. Patients’ follow-up were performed by contacting with patients or their family through mobile phone.

### Statistical analyses

Analyses were performed with the *R* programming language (*R* Core Team, online, 2021; version 4.1.0). Mean ± standard deviation (SD) was used to present continuous normal distributed variables, median (Interquartile Range, IQR) was used to present non-normal distributed parameters. The student *t*-test was applied to the comparison of normal distribution random variables. Wilcoxon signed-rank test was applied to comparison non-normal distribution variables. Besides, a Chi-square test and fisher exact test were employed for comparing categorical data. First, multiply-imputed by chained equations was conducted to impute covariates by using the “mice” package in R. Second, the method least absolute shrinkage and selection operator (LASSO) was done to avoid overfitting by using the “glmnet” package, and we tuned lambda (λ) by a tenfold cross-validation (CV) method by using the “cv.glmnet” function from the “glmnet” R package. Then, the Cox regression analysis was uesd to assess the significance of remained predicted factors in mortality by using the function “coxph” in the R package “survival”, and the prognostic nomogram was established by multivariable Cox regression coefficients based on package “rms”. Finally, the calibration plot of internal validation was conducted via a bootstrap method with 1000 resamples, by the “rms” R package, specifying the parameter “method = “boot”, B = 1000”, from the training set (*n* = 1000). The predicted performance of the established nomogram and the ILD-GAP model was compared with Harrell’s C index (“survival” R package), likelihood ratio testing (“lrtest” function in R package “lmtest”), a continuous version of the net reclassification improvement (NRI) and integrated discrimination improvement (IDI) (R package “survC1” and “survIDINRI”). Additionally, the decision curve analysis (DCA) was performed using the source file “stdca.R”. *P* -values (*P*) less than 0.050 were considered statistically significant.

## Results

### Patient characteristics

**Fig. 1:**
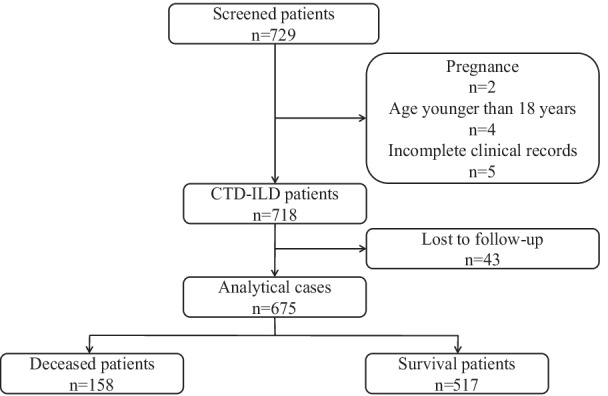
The flowchart of patient screening and selection for this study. CTD-ILD, connective tissue disease-associated interstitial lung disease

The process of patient screening is illustrated in Fig. 1. After excluding the patients with younger than 18 years (n = 4), pregnancy (n = 2), much missing data (n = 5) and loss of follow-up (n = 43), a total of 675 patients eventually entered into the study. There were no significant deviations between the enrolled patients and patients were lost to follow-up in age, gender, occupation, smoking history, days of symptoms, medication treatment history, chronic disease history, pulmonary function test (PFTs) and HRCT (all *P* > 0.050). Therefore, excluding the patients with loss of follow-up may not affect the overall results in our study (Table 1).

**Table 1:** Clinical characteristics of CTD-ILD patients

Characteristics	Lost to follow-up		All		P* value		Patients survived		Patients died	*P*^#^ value	
No. of patients	N = 43		N = 675				N = 517		N = 158		
Age, years	58 ± 11		54 ± 12		0.07		52.7 ± 12.1		59.1 ± 12.0	< 0.001	
Male, n (%)	10 (23.3)		160 (23.7)		1		112 (21.7)		48 (30.4)	0.032	
Farmer, n (%)	25 (58.1)		360 (53.3)		0.649		261 (50.5)		99 (62.7)	0.010	
Ever smoker, n (%)	2 (4.7)		75 (11.1)		0.283		48 (9.3)		27 (17.1)	0.010	
CTD types, n (%)											
PM/DM	5 (11.6)		201 (29.8)		0.017		162 (31.3)		39 (24.7)	0.133	
SS	8 (18.6)		97 (14.4)		0.590		77 (14.9)		20 (12.7)	0.568	
SSc	7 (16.3)		90 (13.3)		0.751		71 (13.7)		19 (12.0)	0.675	
SLE	5 (11.6)		56 (8.3)		0.633		40 (7.7)		16 (10.1)	0.431	
RA	6 (14.0)		50 (7.4)		< 0.001		25 (4.8)		25 (15.8)	< 0.001	
AS	0 (0)		1 (0.1)		1		1 (0.2)		0 (0)	1	
MCTD	0 (0)		40 (5.9)		0.194		31 (6.0)		9 (5.7)	1	
UCTD	11 (25.6)		103 (15.3)		0.114		81 (15.7)		22 (13.9)	0.684	
OCTD	1 (2.3)		37 (5.5)		0.586		29 (5.6)		8 (5.1)	1	
Other complications, n (%)											
Hypertension	11 (25.6)		146 (21.6)		0.676		110 (21.5)		36 (22.8)		0.770
Diabetes	2 (4.7)		58 (8.6)		0.534		46 (8.9)		12 (7.6)		0.727
Days of symptoms, months	6 (2–24)		9 (2–36)		0.054		6 (2–24)		9 (2–36)		0.054
Baseline lung function											
FVC, %Predicted	78.3 ± 15.9		80.2 ± 20.6		0.363		80.5 ± 20.1		79.3 ± 22.0		0.553
FEV_1_, %Predicted	80.0 ± 14.3		79.8 ± 21.5		0.940		80.0 ± 21.1		78.8 ± 22.9		0.556
FEV_1_/FVC, %	85.2 ± 8.4		82.4 ± 10.3		0.196		82.6 ± 9.4		81.7 ± 12.8		0.384
DLco, %Predicted	60.7 ± 13.6		52.6 ± 20.8		0.076		54.7 ± 20.2		45.9 ± 21.5		< 0.001
Echocardiography											
RAA, cm^2^	12.9 ± 1.8		13.2 ± 3.4		0.398		12.6 ± 2.1		15.1 ± 5.4		< 0.001
LVEF, %	63.4 ± 2.9		63.0 ± 4.0		0.366		63.3 ± 3.5		61.8 ± 5.3		0.001
PASP, mmHg	28.6 ± 15.9		27.6 ± 11.5		0.781		26.1 ± 7.7		32.4 ± 18.5		< 0.001
Image charatern, n (%)											
Honeycombing	3 (7.0)		53 (7.9)		1		30 (6.8)		23 (14.6)		0.001
Fine reticular opacities	14 (32.6)		223 (33.0)		1		160 (30.9)		63 (39.9)		0.046
Diffuse bilateral ground-glass opacities	39 (90.7)		623 (92.3)		0.932		477 (92.3)		146 (92.4)		1
Local Pleural thickening	24 (55.8)		369 (54.7)		0.748		268 (51.8)		101 (63.9)		0.010
Pulmonary bullous	9 (20.9)		108 (16.0)		0.525		74 (14.3)		34 (21.5)		0.042
Hydrothorax	3 (7.0)		75 (11.1)		0.554		47 (9.1)		28 (17.7)		0.004
Hydropericardium	3 (7.0)		82 (12.1)		0.439		52 (10.1)		30 (19.0)		0.004
Small pulmonary nodules	5 (11.6)		137 (20.3)		0.289		111 (21.5)		26 (16.5)		0.208
Treatment, n (%)											
Immunosuppressive agents	20 (46.5)		364 (53.9)		0.431		303 (58.6)		61 (38.6)		< 0.001
Glucocorticoids	41 (95.3)		608 (90.1)		0.384		464 (89.7)		144 (91.1)		0.719
Pirfenidone	0 (0.0)		61 (9.0)		0.075		48 (9.3)		13 (8.2)		0.805
Follow-up time, months			50 (38–65)				56 (44–69)		20.5 (4–38)		< 0.001

*CTD* connective tissue disease, *PM/DM* polymyositis/dermatomyositis, *SS* sjogren syndrome, *SSc* systemic sclerosis, *RA* rheumatoid arthritis, *SLE* systemic lupus erythematosus, *AS* ankylosing spondylitis, *MCTD* mixed connective tissue disease, *UCTD* undifferentiated connective tissue disease, *OCTD* overlap syndromes, *FVC* forced vital capacity, *FEV*_*1*_ forced expiratory volume in one second, *DLco* carbon monoxide diffusion capacity, *RAA* right atrial area, *LVEF* left ventricular ejection fraction, *PASP* pulmonary artery systolic pressure

*Comparison of the performance of lost to follow-up patients and all analytical patients for clinical characteristics

^**#**^Comparison of the performance of survival patients and deceased patients for clinical characteristics

Data are presented as means ± SD, numbers (%) or median (Interquartile Range)

**Fig. 2:**
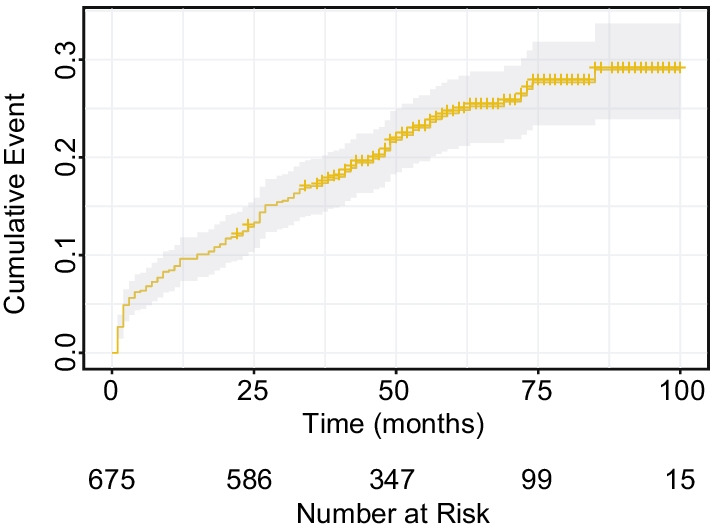
All-cause mortality among 675 Chinese CTD-ILD patients. CTD-ILD, connective tissue disease-associated interstitial lung disease

In this study, the mean age of the cases was 54 ± 12 years (23.7% of male and 11.1% of ever smokers), and the median follow-up period was 50 months (interquartile range, 38–65). The disease subtypes comprise mainly polymyositis/dermatomyositis (29.8%), systemic lupus erythematosus (8.3%), systemic sclerosis (13.3%), ankylosing spondylitis (0.1%), sjogren syndrome (14.4%), mixed connective tissue disease (5.9%), rheumatoid arthritis (RA) (7.4%), undifferentiated connective tissue disease (15.3%) and overlap syndromes (5.5%). 158 patients died during the follow-up period, the 3- and 5-year mortality were 17.1% (95% confidence interval (CI) 14.2–19.8%) and 24.5% (95% CI 20.9–28.0%), respectively (Fig. 2). As compared with survival patients, deceased patients were significantly more likely to be older, males, ever smokers, farmers and treated without immunosuppressive drugs (all *P* < 0.050). Patients with RA had the highest mortality compared to the other CTD subtypes (*P* < 0.001). Deceased patients were also more likely to have lower the percent predicted values of diffusion capacity of lung for carbon monoxide (DLco %Predicted) and left ventricular ejection fraction (LVEF), lager right atrial area (RAA), and higher pulmonary artery systolic pressure (PASP) (all *P* < 0.050). In addition, when presenting with honeycombing, fine reticular opacities, local pleural thickening, pulmonary bullous, hydrothorax and hydropericardium on chest HRCT, most CTD-ILD patients are more exposed to the risk of dying (all *P* < 0.050) (Table 1).

### Model derivation

**Fig. 3:**
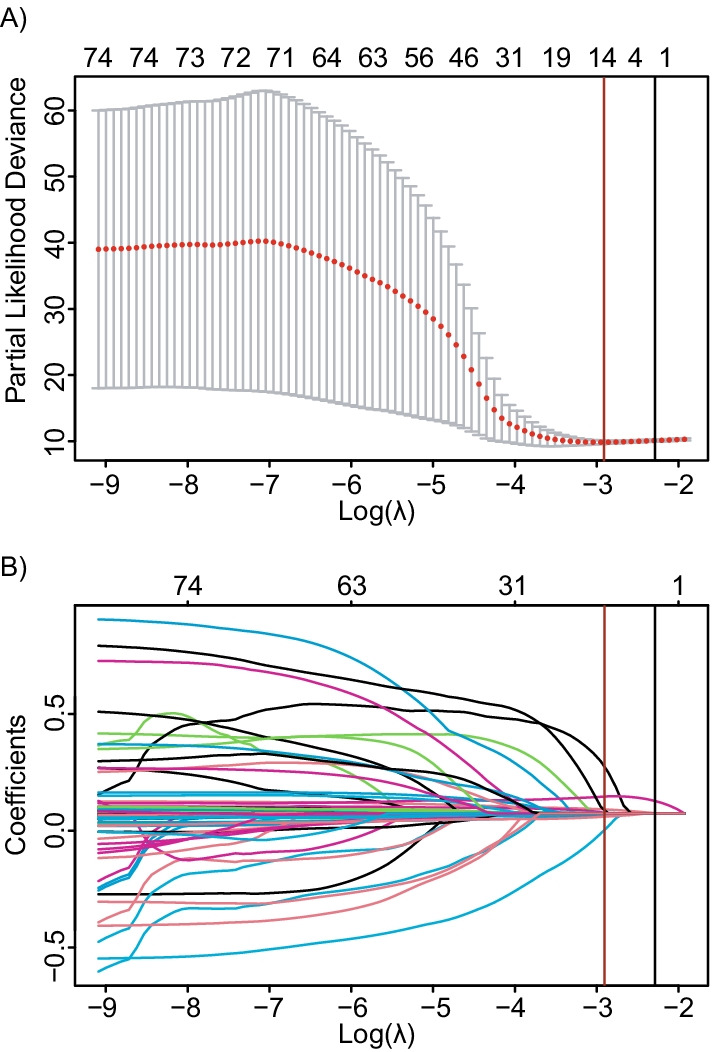
The Cox regression model with LASSO (Least Absolute Shrinkage and Selection Operator) was adopted to reduce the redundancy of high-dimensional features and to select the most useful prognostic features. The lambda with 1 standard error of the minimum criteria (the 1-SE criteria) by the black line, and the red line equals lambda with the minimum criteria. A λ value of 0.052, with log (λ) of − 2.950 was chosen (the minimum criteria) according to tenfold cross-validation (**A**). LASSO coefficient profiles of the 74 features. A coefficient profile plot was produced against the log (λ) sequence. Red vertical line was drawn at the value selected using tenfold cross-validation, where optimal λ resulted in 14 nonzero coefficients (**B**)

A total of 74 prognostic indicators were included in this study. First, we reduced the dimension and picked the most meaningful prognostic indicators by LASSO Cox regression penalty. Subsequently, a tenfold cross-validation of the lasso model was performed for tuning parameter selection via the minimum criteria (Fig. 3A). The trajectory of each prognostic indicators coefficient was observed in the LASSO coefficient profiles with the changing of the log-transformed lambda in LASSO algorithm (Fig. 3B).

**Fig. 4:**
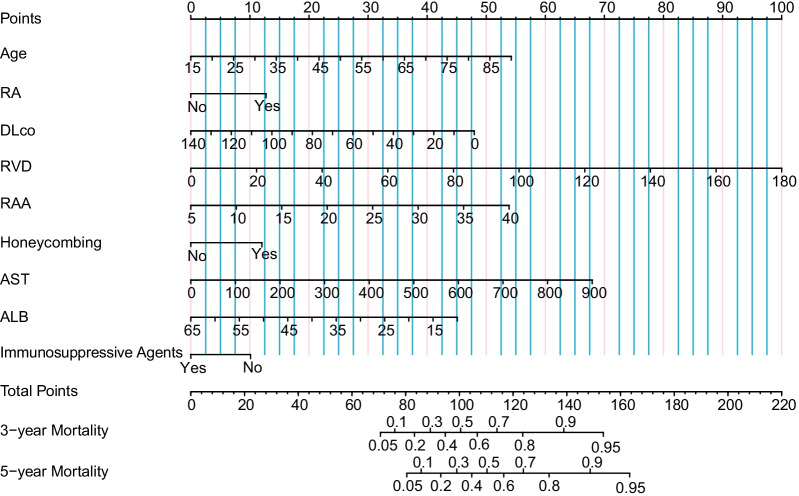
Nomogram predicting CTD-ILD mortality at 3 and 5 years. The nomogram was developed in the primary cohort, with age, rheumatoid arthritis (RA), the percent predicted values of diffusion capacity of lung for carbon monoxide (DLco %Predicted), right ventricular diameter (RVD), right atrial area (RAA), honeycombing, aspartate transaminase (AST), albumin (ALB) and immunosuppressive agents incorporated. The predicted mortality at 3 and 5 years is then obtained from each scale by referring to the corresponding value

Finally, the optimal lambda value was 0.052 (log (lambda) was − 2.950) by using the LASSO algorithm and 14 variables were selected as potential prognosis-related indicators, including age, RA, Dlco %Predicted, right ventricular diameter (RVD), RAA, PASP, LVEF, honeycombing, C-reactive protein (CRP), B-type natriuretic peptide (BNP), aspartate transaminase (AST), γ-Glutamyltranspeptidase (GGT), albumin (ALB) and immunosuppressive agents. Univariable analysis showed that increased age (hazard ratio (HR) 1.041, 95% CI 1.027–1.055), RVD (HR 1.027, 95% CI 1.017–1.038), RAA (HR 1.122, 95% CI 1.093–1.151), PASP (HR 1.025, 95% CI 1.017–1.034), CRP (HR 1.005, 95% CI 1.002–1.008), BNP (HR 1.000, 95% CI 1.000–1.000), AST (HR 1.003, 95% CI 1.002–1.1005), GGT (HR 1.002, 95% CI 1.001–1.1003) and a lower DLCO %Predicted (HR 0.982, 95% CI 0.975–0.990), LVEF (HR 0.949, 95% CI 0.926–0.973), ALB levels (HR 0.936, 95% CI 0.914–0.959) correlated with increased mortality (all *P* < 0.001). Patients with RA (HR 2.292, 95% CI 1.539–3.413, *P* < 0.001) and honeycombing (HR 2.167, 95% CI 1.392–3.373, *P* = 0.001) also had higher mortality. In addition, mortality declined in those patients receiving immunosuppressive agents therapy (HR 0.506, 95% CI 0.367–0.697, *P* < 0.001) (Table 2). Significant variables (*P* value < 0.050) of the univariate analysis were entered into a multivariate Cox model, and showed that age, RA, Dlco %Predicted, RVD, RAA, honeycombinge, immunosuppressive agents, AST, ALB affected overall mortality significantly (all *P* < 0.050) (Table 2). According to multivariable Cox regression analysis, 9 independent variables were enrolled in nomogram for prognostic assessment (Fig. 4).

**Table 2:** Risk factors for all-cause mortality in CTD-ILD

Variables	Unadjusted hazard ratio		Multivariable analysis	
	Hazard ratio (95% CI)	*P*-value	Hazard ratio (95% CI)	*P*-value
Age, years	1.041 (1.027–1.055)	< 0.001	1.035 (1.020–1.050)	< 0.001
Subtypes of CTD				
RA	2.292 (1.539–3.413)	< 0.001	1.788 (1.162–2.750)	0.008
Baseline lung function				
DLco, %Predicted	0.982 (0.975–0.990)	< 0.001	0.984 (0.977–0.992)	< 0.001
Echocardiography				
RVD, mm	1.027 (1.017–1.038)	< 0.001	1.026 (1.012–1.039)	< 0.001
RAA, cm^2^	1.122 (1.093–1.151)	< 0.001	1.058 (1.015–1.102)	0.007
PASP, mmHg	1.025 (1.017–1.034)	< 0.001	1.008 (0.994–1.023)	0.275
LVEF, %	0.949 (0.926–0.973)	< 0.001	0.971 (0.943–1.001)	0.054
Image charatern				
Honeycombing	2.167 (1.392–3.373)	0.001	1.847 (1.158–2.947)	0.010
Treatment				
Immunosuppressive agents	0.506 (0.367–0.697)	< 0.001	0.631 (0.450–0.885)	0.008
Serologic test				
CRP, mg/l	1.005 (1.002–1.008)	< 0.001	1.002 (0.998–1.006)	0.231
BNP, pg/ml	1.000 (1.000–1.000)	< 0.001	1.000 (1.000–1.000)	0.792
AST, U/l	1.003 (1.002–1.005)	< 0.001	1.003 (1.002–1.005)	< 0.001
GGT, U/l	1.002 (1.001–1.003)	< 0.001	1.001 (1.000–1.002)	0.129
ALB, g/l	0.936 (0.914–0.959)	< 0.001	0.959 (0.932–0.987)	0.004
ILD-GAP model	1.413 (1.285–1.554)	< 0.001		

*CTD* connective tissue disease, *RA* rheumatoid arthritis, *DLco* carbon monoxide diffusion capacity, *RVD* right ventricular diameter, *RAA* right atrial area, *PASP* pulmonary artery systolic pressure, *LVEF* left ventricular ejection fraction, *CRP* C-reactive protein, *BNP* B-type natriuretic peptide, *AST* aspartate transaminase, *GGT* γ-Glutamyltranspeptidase, *ALB* albumin

### Model validation

**Fig. 5:**
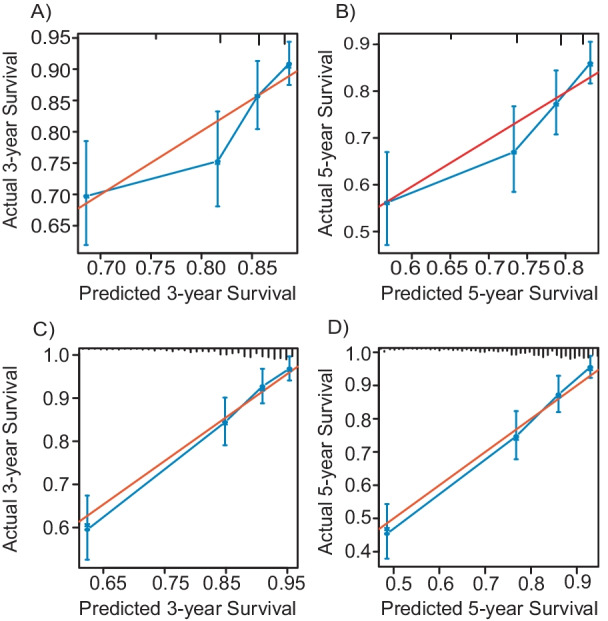
Calibration plots of ILD-GAP model and nomogram showing predicted 3-year (**A** and **C,** respectively) and 5-year (**B** and **D,** respectively) survival by stage against actual survival

The ILD-GAP model exhibited increasing mortality rates in patents with higher scores by univariate variable Cox regression (HR 1.413, 95% CI 1.285–1.554, *P* < 0.001; Table 2). However, the ILD-GAP model did not perform well in predicting mortality (Harrell’s C index 0.652), and calibration plots showed that 3- and 5-year predicted survival rates were overestimated (Fig. 5A, B).

**Fig. 6:**
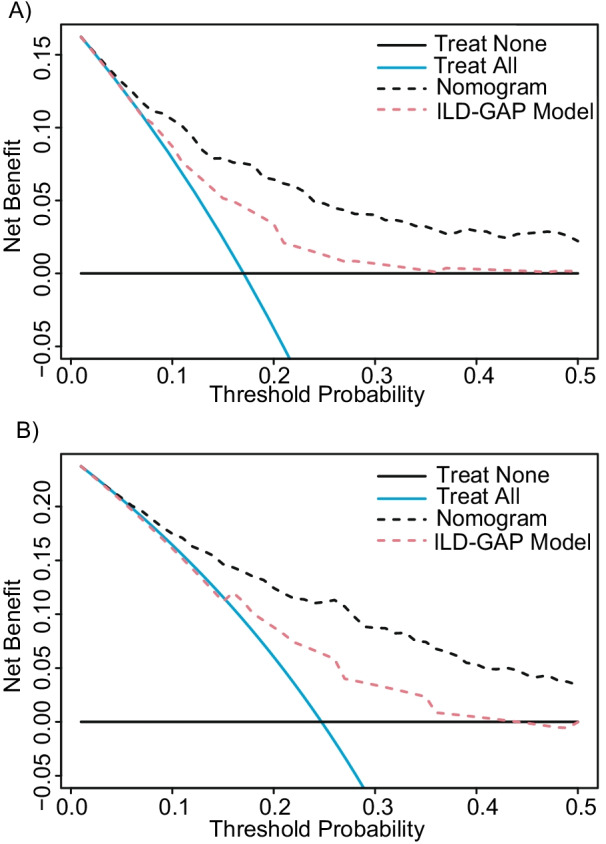
Decision curve analysis comparing the clinical performance of nomogram and the ILD-GAP model. For risk of 3‑year (**A**) and 5-year (**B**) mortality, nomogram showed the highest net benefit for all potential thresholds. The black dot line represents the nomogram and the red dot line represents the ILD-GAP model. The black solid line represents the assumption that no patients have received treatment and the blue solid line represents the assumption that all patients have received treatment

The nomogram exhibited a better prognostic performance (Harrell’s C index 0.767) compared with the ILD-GAP model, because likelihood-ratio test indicated that there was a statistically significant improvement after the inclusion of nomogram in the ILD-GAP model (*P* < 0.001), but no statistical difference after the inclusion of the ILD-GAP model in nomogram (*P* = 0.455) (Table 3). Calibration plots for nomogram predicted 3- and 5-year overall survival showed good agreement with actual observations (Fig. 5C, D). The nomogram also improved the ability of discriminate 3-year (0.137 and 0.294, IDI and NRI respectively, all *P* < 0.001) and 5-year (0.136 and 0.325, IDI and NRI respectively, all *P* < 0.001) mortality rates compared to ILD-GAP model (Table 4). To substantiate the utility of the both models, we performed decision curve analysis. For the optimal decision threshold > 0%, the nomogram showed a better net benefit than the ILD-GAP model for clinical intervention (Fig. 6A, B). In internal validation, the average Harrell’s C index for the prediction models developed in the bootstrap sample was 0.876, and the estimate of optimism was − 0.108.

**Table 3:** Comparison of nomogram and the ILD-GAP model

	Nomogram	ILD-GAP model	Nomogram + ILD-GAPmodel
Likelihood ratio	156.78	48.39	157.34
*P*-value	0.455*	< 0.001^#^	

*Comparison of the performance of predicting overall mortality by using nomogram only and the combination of the nomogram and ILD-GAP model

^#^Comparison of the performance of predicting overall mortality by using ILD-GAP model only and the combination of the nomogram and ILD-GAP model

**Table 4:** Prediction improvement with nomogram compared to ILD-GAP model

	IDI (95% CI)	*P*-value	NRI-continuous (95% CI)	*P*-value
3-year mortality	0.137 (0.092–0.184)	< 0.001	0.294 (0.174–0.417)	< 0.001
5-year mortality	0.136 (0.091–0.182)	< 0.001	0.325 (0.201–0.431)	< 0.001

*IDI* integrated discrimination improvement, *NRI* net reclassification improvement

## Discussion

The ILD-GAP model was derived and validated in a Western cohort but has not been validated in Chinese population to date, its ability to accurately define disease stage is partly debated [14–16]. In order to eliminate potently racial bias from the ILD-GAP model, we developed a nomogram for predicting 3‑ and 5-year mortality of Chinese CTD-ILD patients by using a machine learning approach and tested whether the combination of the nomogram and ILD-GAP model could generate a superior prognostic performance.

Multivariable analysis demonstrated that older age, RA, honeycombing, lower Dlco %Predicted and ALB, increased RVD, RAA and AST associated with higher mortality, but receiving immunosuppressive agents therapy correlated with reduced mortality. These independent risk factors can be supported by previous studies and theories. Age has been demonstrated to be an independent predictor of mortality in CTD-ILD by previous study, because older patients generally have more comorbidities and worse health status [16]. Among ILD, presenting usual interstitial pneumonia (UIP) on chest HRCT has a poor response to corticosteroids and a worse prognosis than other subtypes [17, 18]. Honeycombing occurs in up to 90% of UIP cases, and it is the most specific finding of UIP on chest HRCT [19]. Therefore, honeycombing is correlated with the prognosis of CTD-ILD patients to some extent. Gas exchange impairment is a common pathophysiological change at early stage of ILD, it typically presents as reduction of Dlco [20]. Qiang Fu et al. reported that the percent predicted values of Dlco < 45% is a risk factor for CTD-ILD prognosis [21]. A serum AST elevation and abnormal ALB can be caused by impaired heart, liver and kidney function due to CTD-ILD [22]. Long-term monitoring of serum AST and ALB can be and early warning signal before organ dysfunction occurs. Furthermore, the abnormal increase in AST and hypoalbuminemia have been shown to increase mortality in CTD-ILD patients [23–25]. Long-term hypoxia caused by gas exchange impairment may lead to an increase in pulmonary artery pressure and right ventricular afterload [26]. Right heart enlargement due to persistently increased afterload is a common cause of mortality in patients with ILD which is characterized by the increase of RVD and RAA [27]. In addition, glucocorticoid and immunosuppressive therapy are essential choices for CTD-ILD patients, and mortality can be reduced by the appropriate use immunosuppressive agents [2, 28–30]. ILD can complicate RA and it is associated with an excess in mortality [31]. Research has shown that nearly 10% of RA patient deaths were attributable to ILD. RA patients are more likely to die due to ILD compared to other CTD patients [2, 32].

We developed a nomogram by these independent mortality risk factors based on the multivariable analysis. In this nomogram, we assessed the association between predictor variables and time-to-event outcomes by LASSO-Cox method. Lasso is a machine learning algorithm that utilizes regularization to improve the estimation accuracy, it incorporates an L1-penalization term into the loss function forcing, which can shrink coefficients towards zero. Recently, LASSO-Cox method is popular by researchers, it could minimize overfitting and select predictors of nomogram [33].

In our cohort, the nomogram for Chinese CTD-ILD patients showed better discriminative ability, calibration and clinical net benefit compared with the ILD-GAP model. Despite the combination of the nomogram and ILD-GAP model was found to improve prognostic performance compared with the ILD-GAP model, it could not improve prognostic performance compared with the nomogram. Specifically, Harrell’s C index and calibration curve of the nomogram showed a good concordance for prediction and actual mortality risk. The nomogram also improved the ability of discriminating mortality compared to ILD-GAP model confirmed by integrated discrimination improvement and net reclassification improvement. For decision threshold > 0%, the nomogram showed a higher net benefit than the ILD-GAP model for clinical intervention in decision curve analysis. There are two results might explain why the ILD-GAP model is inferior to the nomogram in predicting prognosis of Chinese CTD-ILD patients. First, the ILD-GAP model was derived and validated in a Western cohort, there was no Chinese population involved. Thus, the risk of bias incurred from ethnic differences should also be considered. Second, the GAP risk prediction model was specifically developed for idiopathic pulmonary fibrosis (IPF) patients to prognosis prediction, from which the ILD-GAP model derived. However, the median survival time of IPF was much shorter compared to CTD-ILD [5, 34]. It is undeniable that the ILD-GAP model can provide important value for the treatment of CTD-ILD patients. To achieve the better predictable results, complex model seems necessary [35–37]. The clinical indicators included in this nomogram were routine and easily acquired data for most hospital which makes it applicable for daily clinical use. We strongly believe that the nomogram could be widely clinical referenced after cross-sectional and longitudinal validation and improvement.

Our study featured some limitations. First, the nomogram was not subjected to external validation, therefore caution is advised when employing it in a clinical framework. To the best of our knowledge, this is the first predictive model developed for predicting all-cause mortality of the Chinese population with CTD-ILD, we believe that an early report is urgent to provide a basis for future studies. Second, the disease categories were included as predictors in the nomogram instead of the serologic autoantibodies, because of the risk of collinearity. Third, The median survival time for CTD-ILD patients was reported to be around 6.5 years, but the median follow-up period was 50 (interquartile range, 38–65) months in our cohort. However, our study had a greater sample size and longer follow-up period than most of previous studies. Fourth, the nomogram and the ILD-GAP model were established by baseline characteristics, and longitudinal disease activity was not considered. Thus, omitted risk-associated trajectories of disease would likely have led to an underestimate of the true relation between CTD-ILD and mortality by the two above-mentioned models.

## Conclusions

In conclusion, the ILD-GAP model performed poorly in predicted mortality of the Chinese patients with CTD-ILD. Our study developed a nomogram for predicting 3‑ and 5-year mortality of Chinese CTD-ILD patients by using a machine learning approach and performed well in predicting mortality risk.

## Data Availability

The datasets used and/or analysed during the current study are available from the corresponding author on reasonable request.

## Abbreviations

CTD: Connective tissue disease
ILD: Interstitial lung disease
CTD-ILD: Connective tissue disease-associated interstitial lung disease
PFTs: Pulmonary function test
PM/DM: Polymyositis/Dermatomyositis
SLE: Systemic lupus erythematosus
SSc: Systemic sclerosis
AS: Ankylosing spondylitis
SS: Sjogren syndrome
MCTD: Mixed connective tissue disease
RA: Rheumatoid arthritis
UCTD: Undifferentiated connective tissue disease
OCTD: Overlap syndromes
HRCT: High-resolution computed tomography
FVC %Predicted: Percent predicted values of forced vital capacity
FEV1%Predicted: Percent predicted values of forced expiratory volume in one second
DLco %Predicted: Percent predicted values of diffusion capacity of lung for carbon monoxide
RVD: Right ventricular diameter
RAA: Right atrial area
PASP: Pulmonary artery systolic pressure
LVEF: Left ventricular ejection fraction
BNP: B-type natriuretic peptide
AST: Aspartate transaminase
GGT: γ-Glutamyltranspeptidase
ALB: Albumin
SD: Standard deviation
IQR: Interquartile range
LASSO: Least absolute shrinkage and selection operator
NRI: Net reclassification improvement
IDI: Integrated discrimination improvement
DCA: Decision curve analysis
